# Event and Comment

**Published:** 1914-07-15

**Authors:** 


// DENTAL REGISTER.

Vol. LXVIII. July 15, 1914. No. 7

### EVENT AND COMMENT.

THE SEASON'S ACTIVITIES. This is the time of the year when the newly graduated dentist is much in evidence and the dental convention is flourishing in almost every state and district. It is very confusing to read the announcements and programmes, and one wonders how so many activities can be carried on by busy dentists, and sometimes when we get the results and begin to figure the costs, we wonder if it pays. Does it pay the young man to spend fifteen hundred to two thousand dollars and give three years of time at the very beginning of the productive period of his life to prepare himself to practice dentistry? If he practices dentistry as a means of making a living for himself and his family, wholly with no higher ideals, we should say no with considerable emphasis. He could do as much or better it may be, by cutting wood, selling sewing machines or automobiles, or even by organizing a bank or insurance company. If, however, a man spends his time and money with the idea that there is a human need that he can supply in the practice of dentistry, and that he is willing to give his life to such a work with an enthusiasm and earnestness that will justify the sacrifices of time and money, then we believe he can safely rest contented in mind as to whether he is called to a professional career, and he will not be disappointed either in professional or material results. It is the fact that so large a number of the men who have taken up dentistry as a calling or life work have found in it such great opportunities for the highest ideals in service, that there has developed an esprit do corps among dentists that compels the dental convention, and it has become necessary as a kind of clearing house for the exchange of ideas, and for the communion of kindred minds. The motive which prompts men to spend time and money to prepare material in the way of papers, clinics, etc., for dental society meetings is not selfish, or it would have no force and there would soon be a decline of interest in these gatherings. The fact that the convention idea seems to be growing constantly in favor, and that never in dental history has there been so much activity manifested in this way, is conclusive evidence of the unselfishness and altruism of our calling when practiced legitimately. In other words the get together spirit is one of the most convincing evidences that we are in fact entitled to recognition as a part, at least, of the greatest of all the noble professions, medicine. Like surgery we have had to develop along the technical lines, but there are abundant signs that we shall have in the future a large share in disease prevention as well as in the rescue and repair work of the past.

NEW YORK'S NARCOTIC LAW. Through the influence of persons who are actively interested in the prevention of the abuse of narcotic drugs by persons having formed this pernicious habit, a law has just been passed which makes it necessary to have a prescription from a physician, dentist or veterinary surgeon before one can purchase from a drug store opium or chloral, or any of their preparations in the State of New York. While it does seem that some restrictions ought to be placed on the indiscriminate use of these drugs, their use in proper preparations and dosage has become so well known that some of them seem like household remedies and their restricted use may at times work great hardship. What household for instance does not know the value and proper use of paragoric. Curiously enough, however, this law permits druggists to sell over their counters, unrestricted, patent medicines which contain these drugs in dangerous quantities and in most cases the purchasers do not know they are buying and taking dangerous and poisonous medicines. It is a perfectly legitimate function for the State to legally protect its people by proper police and health laws, but it seems to us that our National Government ought to go further with its pure food laws, and conserve the public health at least to the extent of making it compulsory that all patent medicines that are inimical to health should be scientifically censored.

SOUTHERN BRANCH OF THE NATIONAL DENTAL ASSOCIATION DISCONTINUES. The Southern Dental Association was organized at Atlanta, July 28th, 1869, with forty-six members from eight or nine Southern States. August 5, 1897, at Old Point Comfort, it consolidated with the American Dental Association as the Southern Branch. It has held separate meetings up to this year, when it decided to discontinue its organization and allow the State Societies of which it was composed to enter the new National as component societies. This was done at Atlanta, June 5, 1914. The Georgia State Society immediately voted unanimously to become a member, with its entire membership, of the National. This organization was undoubtedly first conceived as a result of sectional prejudice, and at one time it had as large a membership as the American. As there is now no North or South, why not a National Association?

